# Mean compression ratio of a self-expandable valve is associated with the need for pacemaker implantation after transcatheter aortic valve replacement

**DOI:** 10.1186/s40001-023-01070-1

**Published:** 2024-01-29

**Authors:** Yiming Qi, Yuefan Ding, Wenzhi Pan, Xiaochun Zhang, Xiaolei Lin, Shasha Chen, Lei Zhang, Daxin Zhou, Junbo Ge

**Affiliations:** 1 Department of Cardiology, Zhongshan Hospital, Shanghai Institute of Cardiovascular Diseases, National Clinical Research Center for Interventional Medicine, Fudan University, Shanghai, China; 2 School of Data Science, Fudan University, Shanghai, China

**Keywords:** TAVR, Self-expandable valve, Pacemaker implantation, Compression level, Mechanical stress

## Abstract

**Background:**

The risk and timing of permanent pacemaker implantation (PPMI) after transcatheter aortic valve replacement (TAVR) is still hard to predict. We aimed to analyze the relationship between the compression ratio of a self-expandable valve (SEV) and the need for PPMI after TAVR.

**Methods:**

A total of 106 patients who were implanted with the VitaFlow transcatheter aortic valve system and for whom complete imaging information was available were included in this retrospective cohort study. Eight lines perpendicular to the long axis of the SEV were drawn (the top and bottom of the SEV and the intersection of each row of wires) for measurement purposes. The compression ratio was calculated as 1 − (in vivo meridian/in vitro meridian) and compared between patients undergoing and those not undergoing PPMI after adjusting for implantation depth. Multivariable logistic regression and Cox proportional hazards models were used to assess factors associated with the risk and timing of the need for PPMI.

**Results:**

Fifteen (14.2%) patients underwent PPMI after TAVR. Patients with a higher mean compression ratio (20%, odds ratio [OR] = 214.82; *p* < 0.001) and prior right bundle branch block (OR = 51.77; *p* = 0.015) had a higher risk of the need for PPMI after TAVR. These two factors were also associated with the timing of PPMI, according to the Cox proportional hazards model.

**Conclusions:**

The compression ratio of the SEV was positively associated with the risk of PPMI after TAVR, and the association was most significant in the annular and supravalvular planes. The compression ratio may also affect the time to PPMI.

## Background

Transcatheter aortic valve replacement (TAVR) is a safe and effective treatment for patients with severe aortic stenosis with even low surgical risks [1, 2]. The necessity of permanent pacemaker implantation (PPMI) caused by conduction block remains a common complication after TAVR, which increases mortality among these patients [3]. The anatomical characteristics of the conduction pathway associated with the mechanical stress of the prosthesis are believed to be one of the main mechanisms of the necessity for PPMI after TAVR [4]. The prediction of stress distribution in the tissue around the prosthesis is reportedly beneficial in predicting the risk of the need for PPMI [5, 6]. The incidence of delayed PPMI has increased in recent years, which may be due to early-discharge practice and the resulting sustained stress of the self-expandable valve (SEV) [7].

An SEV, made of memory alloy, returns to its original size at human body temperature, and the pressure it exerts on the surrounding tissue is proportional to the degree of prosthesis compression [8]. Therefore, we thought the compression level of the SEV may associated with the risk of conduction block after TAVR, which may be used to evaluate the risk and timing of PPMI. In this study, we measured the SEV compression ratio with the aim of determining its correlation with the need for PPMI after TAVR.

## Methods

### Study population

This retrospective cohort study was approved by the Ethics Committee of Zhongshan Hospital, Fudan University (No. B2020-039), and written informed consent was obtained from all the participants for participation in this study and publication of their data. A total of 381 patients with aortic stenosis were treated with TAVR at Zhongshan Hospital (a tertiary teaching hospital affiliated with Fudan University) in Shanghai, China from June 2015 to September 2021. We excluded patients with previous PPMI, TAVR, or surgical aortic valve replacement; those who underwent valve-in-valve TAVR during the procedure; those transferred for surgical operation owing to complications; and those who died of any cause. Ultimately, 106 patients who were implanted with the VitaFlow transcatheter aortic valve system (Microport CardioFlow Medtech Corporation, Shanghai, China) and for whom complete imaging information was available were included in this analysis.

### Data acquisition, pre-processing, and endpoint definition

Baseline characteristics, medical history, pre-procedural electrocardiograms, procedural characteristics, pre-procedural transesophageal/transthoracic echocardiograms, laboratory results, and intraoperative X-ray images were acquired for each patient. Percent oversizing of the prosthetic valve was calculated as the ratio of the nominal valve perimeter to the measured aortic annulus perimeter via computed tomography images. The primary endpoint was PPMI within 60 days of TAVR owing to complete heart block (CHB), high-degree atrioventricular block (HAVB), sinus arrest, or symptomatic bradycardia. The secondary endpoint was time to PPMI, defined as the number of days between TAVR and PPMI, censoring data for patients without PPMI. The need for PPMI was evaluated by a consensus committee consisting of experienced cardiac electrophysiology specialists and interventional cardiologists.

### Image processing

**Fig. 1:**
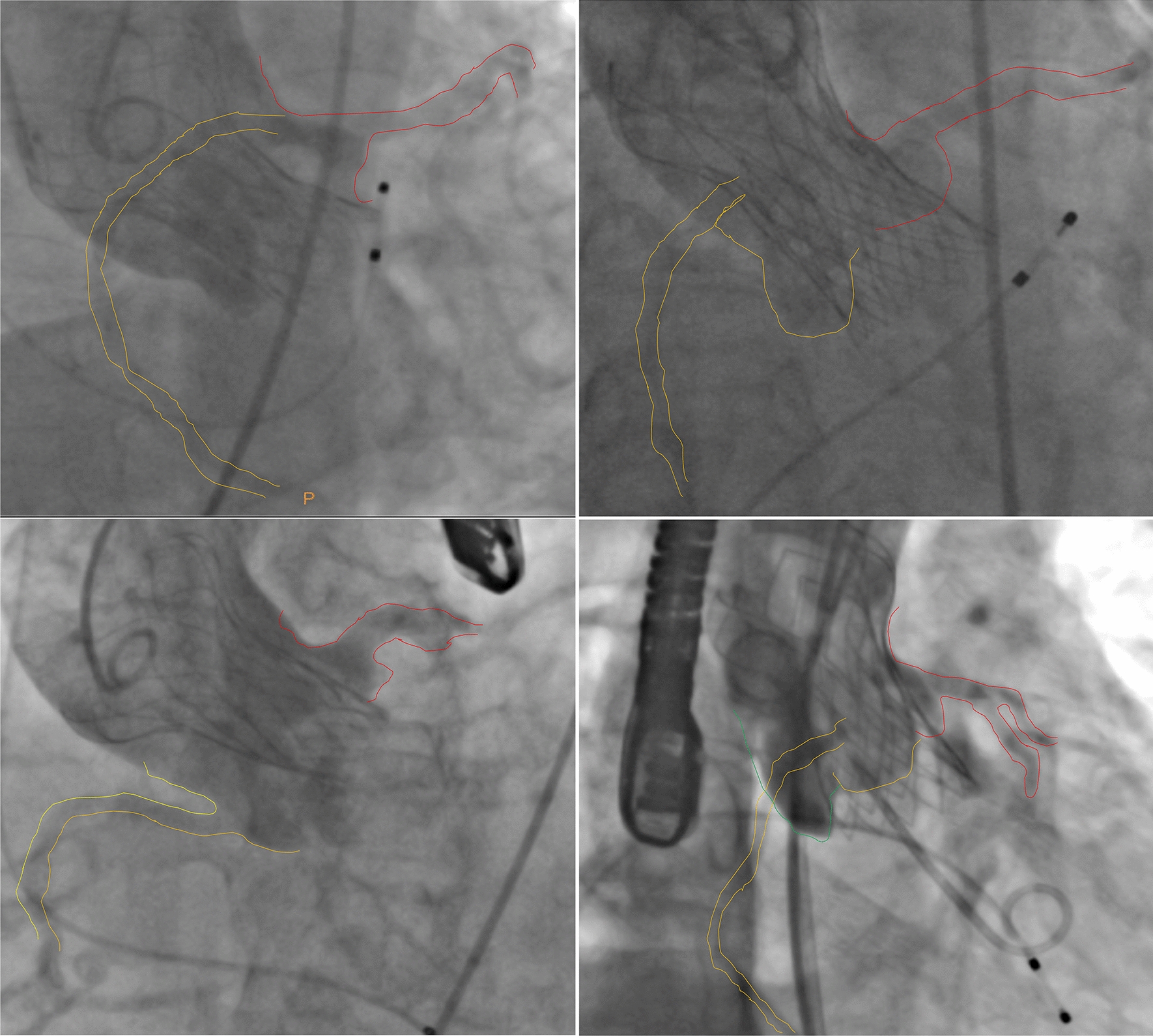
The projection angles were divided into four categories according to the position of the coronary artery and the shape of the sinus. Top left: the left and right coronary sinuses overlap; top right: the right and non-coronary sinuses overlap; bottom left: the non-coronary sinus is in the middle; bottom right: the right coronary sinus is in the middle

**Fig. 2:**
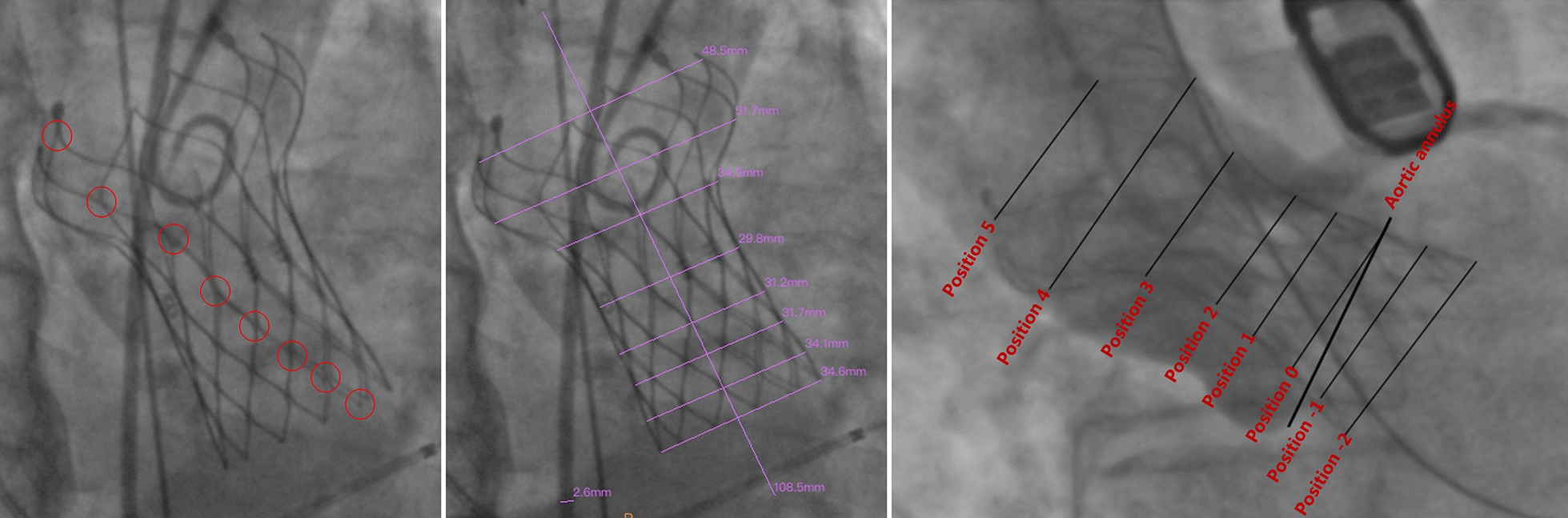
Eight lines perpendicular to the long axis of the self-expandable valve (SEV) drawn (middle) for measurement purposes; these were drawn at the top and bottom of the SEV and at the intersection of each row of wires (left). The line closest to the annulus plane was defined as position 0; those below the annulus plane were positions − 1, − 2, and − 3; and those above the annulus plane were positions 1, 2, 3, and so on

We selected angiographic images of the aortic root after valve implantation; the projection angles were determined according to the position of the coronary artery and the shape of the sinus. The projection angles were divided into four categories, with the following order of priority: the view in which the left and right coronary sinuses overlap, that in which the right and non-coronary sinuses overlap, that in which the non-coronary sinus is in the middle, and that in which the right coronary sinus is in the middle (Fig. 1). The annulus plane was defined as the lowest position of sinus angiography in the selected projection angle. After calibrating the length according to the pigtail catheter, eight lines were drawn perpendicular to the long axis of the SEV (the top and bottom of the SEV and the intersection of each row of wires) for measurement purposes (Fig. 2). We compared the measured meridian with the in vitro measured meridian of the stent (data not shown owing to commercial confidentiality), and we calculated the compression ratio as 1 − (in vivo meridian/in vitro meridian). The line closest to the annulus plane was defined as position 0; those below the annulus plane as positions − 1, − 2, and − 3; and those above the annulus plane as positions 1, 2, 3, and so on (Fig. 2). The mean compression ratio was calculated using the positions most relevant to PPMI.

### Statistical analysis

Continuous variables were described using the mean and standard deviation, whereas categorical variables were described using the frequency and proportion per category. Patient characteristics were compared using a two-sample *t*-test for continuous variables and a Chi-square or Fisher’s exact test for categorical variables. Univariate logistic regression was performed for each position in which compression was measured, the maximum compression value, and the mean compression value for positions 1 to 4. Forward variable selection was performed, and a multivariable logistic regression model was used to determine the association between the factors and the risk of the necessity of PPMI. Time-to-event analysis was performed using a multivariate Cox proportional hazards model to identify potential factors influencing the time to PPMI among patients who underwent TAVR. All statistical analyses were performed using R, version 4.1.2 (R Foundation for Statistical Computing, Vienna, Austria). Daxin Zhou had full access to all the data in the study and takes responsibility for its integrity and the data analysis.

## Results

### Patient characteristics

A total of 106 patients treated with TAVR were included, of whom 15 (14.2%) underwent PPMI. The major reasons for PPMI were CHB (11/15, 73%) and HAVB (4/15, 27%). Twelve patients underwent PPMI within 7 days of TAVR, while 2 and 1 PPMIs were conducted between 8 and 30 days and beyond 30 days, respectively. Patient characteristics are summarized in Table 1. Compared to those not requiring PPMI, patients requiring PPMI had a similar disease history and a higher mean compression ratio (21.4% vs. 8.4%; *p* < 0.001).

**Table 1:** Patient characteristics

	Non-PPMI (*n* = 91)	PPMI (*n* = 15)	Total (*n* = 106)	*p* value
Age	75.39 (6.98)	78.8 (6.59)	75.88 (7.00)	0.081
Female	38 (41.8%)	2 (13.3%)	40 (37.7%)	0.012
CAD	73 (80.2%)	13 (86.7%)	86 (81.1%)	0.527
Hypertension	47 (51.6%)	6 (40%)	53 (50%)	0.420
Smoker	10 (11%)	3 (20%)	13 (12.3%)	0.432
History of stroke	2 (2.2%)	1 (6.7%)	3 (2.8%)	0.523
History of syncope	9 (9.9%)	3 (20%)	12 (11.3%)	0.377
Atrial fibrillation	17 (18.7%)	7 (46.7%)	24 (22.6%)	0.061
Prior RBBB	4 (4.4%)	2 (13.3%)	6 (5.7%)	0.353
BAV	48 (52.7%)	11 (73.3%)	59 (55.7%)	0.137
AVA	0.695 (0.202)	0.775 (0.338)	0.706 (0.226)	0.386
Oversize rate	− 0.008 (0.078)	0.009 (0.049)	− 0.005 (0.074)	0.276
Pre-dilatation	89 (97.8%)	14 (93.3%)	103 (97.2%)	0.899
Post-dilatation	28 (30.8%)	5 (33.3%)	33 (31.1%)	1.000
Mean compression	0.084 (0.082)	0.214 (0.066)	0.103 (0.092)	< 0.001

Values are mean ± SD or frequency (%)

*AVA* aortic valve area, *BAV* bicuspid aortic valve, *CAD* coronary artery disease, *PPMI* permanent pacemaker implantation, *RBBB* right bundle branch block

*P* values are obtained by two-sample *t*-test

### Image measurement

**Fig. 3:**
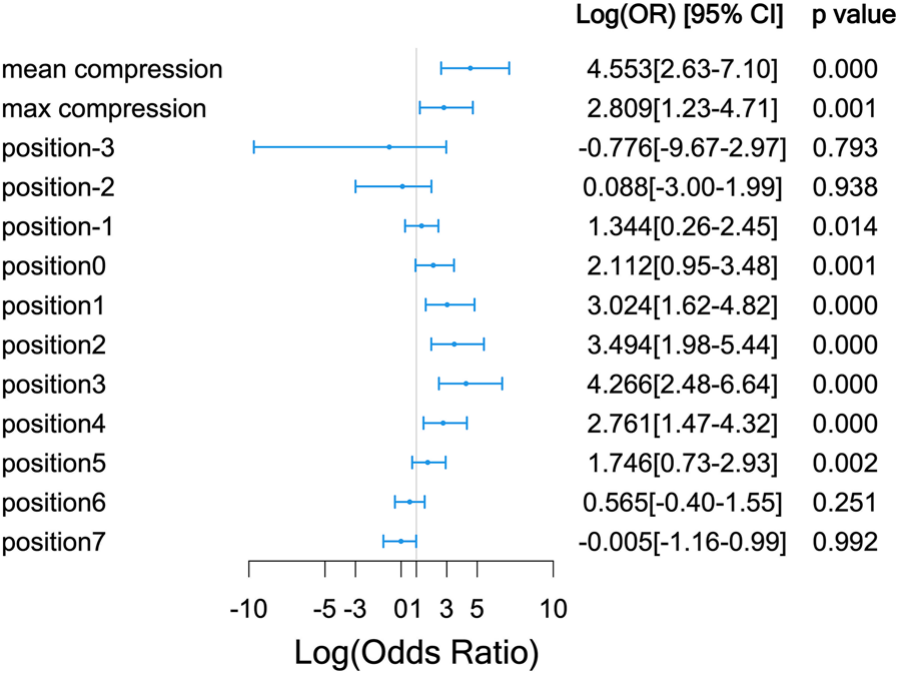
Comparison of the compression ratio at each position of the self-expandable valve

The projection angle in which the left and right coronary sinuses overlapped was selected in 67 cases (63.2%), that in which the right and non-coronary sinuses overlapped was selected in 22 cases (20.8%), that in which the non-coronary sinus was in the middle was selected in 15 cases (14.2%), and that in which the right coronary sinus was in the middle was selected in 2 cases (1.9%). Comparisons of the compression ratios of each position of the SEV are illustrated in Fig. 3; it was highest in patients with a PPMI in positions − 1 to 5. Therefore, the mean compression ratio was calculated using positions 0 to 4.

### Identification of risk factors

**Fig. 4:**
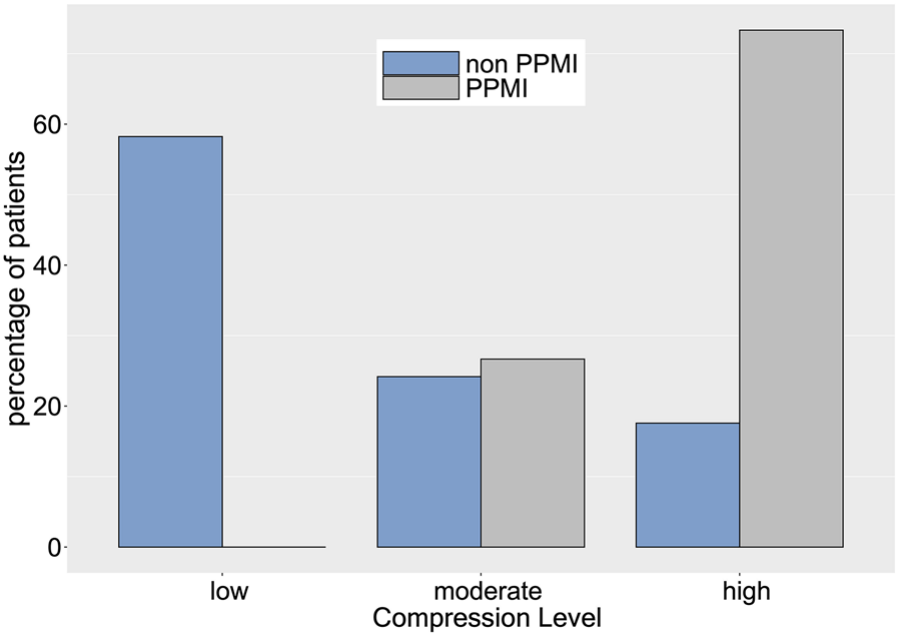
The permanent pacemaker implantation rates among low (≤ 0.09220), moderate (> 0.09220 and ≤ 0.15899), and high (> 0.15899) mean compression ratio groups after transcatheter aortic valve replacement

Candidate risk factors were selected based on their univariate associations with the need for PPMI as well as their clinical significance. The multivariable logistic regression models for the prediction of the need for PPMI are presented in Table 2. The mean compression ratio (20%, odds ratio [OR] = 214.82; *p* < 0.001) and prior right bundle branch block (RBBB; OR = 51.77; *p* < 0.001) were independent risk factors for the need for PPMI. Although atrial fibrillation (OR = 5.08; *p* = 0.054) and a history of smoking (OR = 6.29; *p* = 0.095) did not achieve statistical significance in the model, these should not be discounted as potential risk factors, given the relatively small sample of this study. Patients were divided into three groups according to quantiles of mean compression ratio: low (mean compression ratio ≤ 0.09220), moderate (0.09220 < mean compression ratio ≤ 0.15899), and high (mean compression ratio > 0.15899). The PPMI rates in these three groups are displayed in Fig. 4.

**Table 2:** Multivariate logistic regression models for risk factors of PPMI

	Odds ratio (95% CI)	*p* value
Mean compression (20%)	214.82 (20.53, 5803.48)	< 0.001***
Prior RBBB	51.77 (2.26, 1632.21)	0.015*
Atrial fibrillation	5.08 (1.01, 29.67)	0.054^#^
Smoker	6.29 (0.71, 62.17)	0.095^#^

Abbreviations as in Table 1

Signif. codes: 0 ‘***’ 0.001 ‘**’ 0.01 ‘*’ 0.05 ‘^#^’ 0.1 ‘^†^’ 1

### Time-to-PPMI analysis

**Fig. 5:**
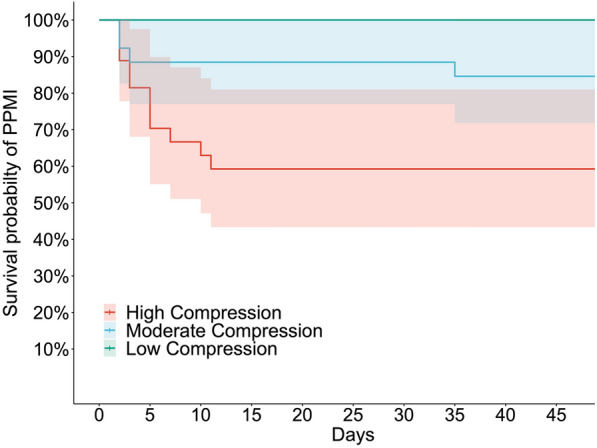
Kaplan–Meier curves of the low, moderate, and high mean compression ratio groups, indicating that patients with a higher mean compression ratio are likely to undergo permanent pacemaker implantation sooner than patients with a lower mean compression ratio (*p* < 0.001)

Time to PPMI and the censoring status (whether the patient underwent PPMI within 60 days of TAVR) were treated as the outcomes in the Cox proportional hazards model, and predictors that potentially influence patients’ time to PPMI are provided in Table 3. In brief, patients with a higher mean compression ratio (hazard ratio [HR] = 53.41; *p* < 0.001), prior RBBB (HR = 39.44; *p* < 0.001), history of smoking (HR = 7.24; p = 0.009), and atrial fibrillation (HR = 3.46; *p* = 0.030) had a shorter time from TAVR to PPMI after adjusting for other factors in the model. Kaplan–Meier curves of the three compression level groups (Fig. 5) revealed that patients with a higher mean compression ratio were more likely to require PPMI sooner than patients with a lower mean compression ratio (*p* < 0.001).

**Table 3:** Cox proportional hazards model for the time to PPMI

Covariances	Hazard ratio (95% CI)	*p* value
Mean compression (20%)	53.41 (11.2, 254.7)	< 0.001***
Prior RBBB	39.44 (5.73, 271.5)	< 0.001***
Smoker	7.24 (1.65, 31.73)	0.009**
Atrial fibrillation	3.46 (1.13, 10.57)	0.030*

Abbreviations as in Table 1

Signif. codes: 0 ‘***’ 0.001 ‘**’ 0.01 ‘*’ 0.05 ‘^#^’ 0.1 ‘^†^’ 1

## Discussion

The need for PPMI due to conduction block remains a major complication after TAVR. Mechanical compression of the surrounding tissue by the prosthesis, especially the vulnerable area near the junction of the right and non-coronary sinus, where the conduction bundle crosses [5, 6], is considered to be one of the main mechanisms of the need for PPMI after TAVR [7]. The rise in the incidence of delayed PPMI in recent years [7] raises new questions regarding the assessment of the need for PPMI and the choice of post-discharge monitoring modalities.

To the best of our knowledge, the pressure distribution of the device landing zone is mainly simulated and predicted via preoperative finite element analysis [9], and there is no evaluation method for the actual pressure after implantation. In a previous study, the vast majority of implanted valve frames suffered from some degree of compression [10]. In vitro experiments on valve stents made of memory alloys demonstrated that, under a constant temperature, the radial support force is proportional to the degree of valve compression [8].

Therefore, we used postoperative fluoroscopic images of the SEV to measure and calculate the compression ratio in different positions of the valve frame. As the implantation depth of the prosthesis was not the same in different patients, and implantation depth affects the need for PPMI [11, 12], we compared the SEV compression ratios at the same location relative to the annulus. We discovered significant differences in positions − 1 to 5 between patients undergoing PPMI and those not undergoing PPMI, with higher rates of compression associated with higher rates of PPMI. Positions − 1 to 5 are equivalent to the middle and lower parts of the valve frame and are mostly located near the annulus plane and supravalvular structures. This is the main anchoring area of the SEV, and the position experiencing the greatest force [13]. We selected positions 0 to 4 (those with the largest differences between the groups) to calculate the average compression ratio for multivariable analysis. The results suggested that the mean compression ratio differed significantly between the PPMI and non-PPMI groups; hence, it is an independent risk factor for PPMI. When we further divided patients into three groups according to the mean compression ratio, we discovered that the compression ratio affected the time to PPMI; a larger compression ratio was associated with earlier PPMI. Therefore, we believe that measurement of the compression ratio in the lower part of the SEV after TAVR may be used to evaluate the risk of the need for PPMI and may be predictive of the late need for PPMI. Owing to the limited sample size, we could not accurately determine the range of compression ratios that indicate that a patient is prone to require late PPMI.

As implantation depth is a risk factor for the need of PPMI [11, 12], the compression ratios at especially positions − 2 and − 3 should differ between patients undergoing and those not undergoing PPMI. However, this was not the case, possibly because there was no significant difference in the implantation depth between the two groups (3.666 ± 2.873 mm vs. 3.664 ± 3.623 mm, *p* = 0.998). The vast majority of SEVs were implanted at a depth of no more than 7.5 mm; position − 2 was approximately equivalent to a depth 7.6–12.5 mm, and position − 3 was approximately equivalent to a depth of 12.5 mm or more. Our results suggest that the compression ratio may be a more sensitive indicator of the need for PPMI than implantation depth when the implantation is not deep.

The vulnerable area of the conduction tract is located in the interventricular septal region at the junction of the right and non-coronary sinuses [7]. Therefore, the compression ratio of the valve frame at the right and non-coronary sinus overlap position may most accurately reflect the force exerted in the vulnerable position. However, angiographic images are normally obtained in the left and right coronary sinus overlap position after implantation, that is, the non-coronary sinus is located at the lowest position, and the view is relatively close to the vulnerable area. Therefore, we prioritized this projection angle in our study. Our results showed that the SEV compression ratio measured at the left and right coronary sinus overlap position or the right and non-coronary sinus overlap position was related to the need for PPMI. However, owing to the limitations of sample size and imaging data, it remains unclear which projection angle is most suitable for the prediction of the need for PPMI.

Other independent risk factors for the need for PPMI in our multivariable analysis were prior RBBB and atrial fibrillation. RBBB has been reported as a risk factor for the need for PPMI in many previous studies [11, 12, 14, 15]. The high incidence of new-onset left bundle branch block after TAVR may explain why prior RBBB is strongly associated with CHB or HAVB [3]. A previous study revealed that atrial fibrillation is associated with the need for PPMI owing to an underlying conduction disease predisposing to both atrial fibrillation and conduction block [16].

Many studies suggest that the oversize rate is related to the need for PPMI [17, 18], but our results were not in agreement (− 0.8% ± 7.8% vs. 0.9% ± 4.9%, *p* = 0.420). This may be owing to the low oversize rate in our study, as downsized prostheses were often selected after balloon pre-dilation because of the large proportion of bicuspid valves (~ 56% of the total cases in our study) and relatively heavy calcification. Although the mean oversize rate was close to zero, the compression ratio differed between groups, suggesting that the compression ratio may be a more accurate indicator of the need for PPMI than the oversize rate. For patients with the same annulus and SEV size, the compression ratio of the prosthesis typically differs owing to different degrees of valve leaf calcification, adhesion, and fusion.

Owing to the differences in metals and shapes used for different SEVs, comparison of different SEVs is challenging. Therefore, we only included patients implanted with the same type of SEV, which is one of the reasons for the small sample. Thus, one should be cautious when applying the results of this study directly to patients implanted with other types of SEVs; however, the measurement method used in this study and its relationship with the need for PPMI should be applicable to all SEVs.

### Study limitations

First, the sample was relatively small; however, this is, to our knowledge, the first report of intraoperative fluoroscopic image-measured compression ratios being significantly correlated with the need for PPMI after TAVR. Studies with larger samples are needed to confirm this conclusion and clarify the dose–effect relationship. Second, owing to the retrospective nature of this study, projection angles with aortic root angiography after implantation were limited, and we were not able to determine the best projection angle for measurement of the compression ratio. Third, owing to the small sample, we could not separately analyze the bicuspid and tricuspid valves, which may result in differences in the location of maximum compression. We are collecting relevant cases and hope to obtain more accurate results in future studies.

## Conclusions

In this study, the compression ratio of the SEV was positively associated with the risk of the need for PPMI after TAVR, and the association was most significant in the annular and supravalvular planes. The compression ratio may also affect the time to PPMI.

## Data Availability

The datasets generated and analyzed during the current study are not publicly available due to the violation of patient privacy and the absence of informed consent for online raw data. Still, they are available from the corresponding author upon reasonable request.

## Abbreviations

CHB: Complete heart block
HAVB: High-degree atrioventricular block
HR: Hazard ratio
OR: Odds ratio
PPMI: Permanent pacemaker implantation
RBBB: Right bundle branch block
SEV: Self-expandable valve
TAVR: Transcatheter aortic valve replacement
